# Chemotherapy of Breast Cancer Cells Using Novel pH-Responsive Cellulose-Based Nanocomposites

**DOI:** 10.15171/apb.2019.015

**Published:** 2019-02-21

**Authors:** Mojtaba Abbasian, Farideh Mahmoodzadeh, Azra khalili, Roya Salehi

**Affiliations:** ^1^Department of Chemistry, Payame Noor University, P.O. BOX: 19395-3697, Tehran, Iran.; ^2^Halal Research Center of IRI, FDA, Tehran, Iran.; ^3^Drug Applied Research Center and Department of Medical Nanotechnology, Faculty of Advanced Medical Science, Tabriz University of Medical Science, Tabriz, Iran.

**Keywords:** Cellulose, Drug delivery systems, pH-responsive, Magnetic and zinc oxide nanocarriers, RAFT polymerization, Breast cancer

## Abstract

***Purpose:*** The objective of the current study was to compare the anticancer efficacy of doxorubicin-loaded cellulose based magnetic (Fe_3_O_4_), zinc oxide (ZnO) nanoparticles on and free doxorubicin (DOX) on MCF-7 breast cancer cells.

***Methods:*** Novel pH-sensitive cellulose-graft poly acrylic acid based Fe_3_O_4_ (Cellulose-g-PAAg- PAcMNPs) and ZnO (Cellulose-g-PAA-g-PAcZnO) nanocomposites were synthesized via polymerization of acrylic acid and modified 3-(trimethoxysilyl) propyl methacrylate onto the cellulosic backbone via reversible addition-fragmentation chain transfer (RAFT) method.

***Results***: Cellulose-g-PAA-g-PAcMNPs and Cellulose-g-PAA-g-PAcZnO nanocarriers with mean diameter of 15 and 38 nm were prepared successfully. DOX was loaded effectively to the ZnO and Fe_3_O_4_ nanocarriers via complexing and electrostatic force with great encapsulation efficiency of 99.07% and 98.92%, respectively. DOX-loaded nanocarriers showed obvious pHdependent tumor specific drug release pattern. MTT assay results indicated that IC50 of the DOX loaded Cellulose-g-PAA-g-PAcZnO, DOX loaded Cellulose-g-PAA-g-PAcMNPs and free DOX after 48 hours treatment with MCF7 cell lines were about 24.03, 49.27 and 99.76 μg mL^−1^, respectively. Therefore both DOX nanoformulations significantly increase antitumor ability compared to free DOX (*P* < 0.05). The results of MTT assay and DAPI staining revealed that DOX-loaded Cellulose-g-PAA-g-PAcZnO NPs show higher chemotherapy efficiency in MCF7 breast cancer cell line compare to the DOX-loaded Cellulose-g-PAA-g-PAcMNPs due to high interaction of ZnO with DOX.

***Conclusion:*** The formation of the complexes between the DOX and ZnO nanoparticles at the chelating sites of the quinone and the phenolic oxygen molecules of DOX, lead to more sustained drug release and enhanced chemotherapy effectiveness by increasing the intracellular concentration of DOX.

## Introduction

Chemotherapy is a common type of treatment for cancer patients.^1,2^ In spite of the effectiveness of anticancer drugs, most of them have problems and causing intense side effects. Doxorubicin (DOX) belonging to the antimetabolite category, leads to myelosuppression and cardiotoxicity when were consumed in cancer therapy. In an attempt to avoid and control the side effects of DOX, nanocarriers with multi-functional features and high advantages like, polymeric nanocomposites, silica, micelles, liposomes, magnetic (Fe_3_O_4)_ and zinc oxide (ZnO) nanoparticles could be convey the drugs to the cancerous tissues safely by the improved retention and permeability effects.^3-6^ The nanocarriers based on synthetic and natural polymers have been investigated for anticancer drug delivery systems (DDSs).^7-9^ A favorable DDS should has the following advantages: sustained and controlled release of drugs in cancerous tissue during cellular uptake via tumor cells, biocompatibility, high stability and long circulation in the bloodstream, and simply conducting drugs to the cancer tissues by active or passive targeting. ^10,11^

DDS based on ZnO and Fe_3_O_4_ nanoparticles have achieved importance in tumor therapy. Fe_3_O_4_ has been greatly explored in cancer research due to high magnetic responses, stable quality and easy achievement as well as, ZnO nanoparticle have achieved importance due to inherent preferential cytotoxicity against cancer cells, easier to produce and its high drug loading efficiency. As a result, synthetic or natural polymers were used as the coating of nanoparticles for enhancement of the therapeutic effect, reduce the toxicity and improve the biodegradability.^12,13^

Cellulose is the most common natural biopolymer and polysaccharide with extensive uses in biotechnological and medicine fields owing to its biodegradability, biocompatibility, non-toxicity, and high chemical modification due to its reactive centers (hydroxyl group) present on its structure. The chemical modification of natural cellulose by graft copolymerization could considerably change its features and has attracted scientists’ attention.^14-19^

Living/controlled polymerization approaches like atom-transfer radical polymerization,^20-22^ reversible addition-fragmentation chain transfer (RAFT) mediated polymerization,^23-25^ and nitroxide mediated polymerization^14,26,27^ can be utilized to chemical modification of cellulosic materials. Among these, RAFT polymerization is especially motivating, mostly because of the production of block copolymers, post-modified polymers, and homopolymers with the controlled molecular weight (narrow dispersity).^28-31^

In this study, we have developed smart nanocarriers based on cellulose with good biocompatibility, pH-controlled DOX release behavior, and efficient anticancer efficacy on breast cancer cells. In this context, cellulose-g-poly acrylic acid (Cellulose-g-PAA) was synthesized through the polymerization of acrylic acid directly onto the propionic acid methyl cellulose xanthate RAFT agent. Then, Fe_3_O_4_ nanocarrier (Cellulose-g-PAA-g-PAcMNPs) synthesized via polymerization of acrylate grafted magnetic nanoparticles (AcMNPs) onto the Cellulose-g-PAA, as well as ZnO nanocarrier (Cellulose-g-PAA-g-ZnO) synthesized via polymerization of acrylate grafted ZnO nanoparticles (AcZnO) onto the Cellulose-g-PAA backbone. Moreover, the anti-cancer drug DOX was loaded effectively to the nanocarriers through electrostatic interactions and complexation with high loading efficiency. Finally, anticancer effects, toxicities, bio-distribution, drug loading properties, and biocompatibility of the nanocarriers were studied.

## Materials and Methods

### Materials

Two-bromopropionic acid 99%, acrylic acid (AA) 99%, ethanol 99.8%, methanol 99.8%, NaOH_,_ 3-(trimethoxysilyl) propyl methacrylate (TSPMA) (99%), 3-(4,5-dimethylthiazol-2-yl)-2,5 diphenyltetrazolium bromide (MTT), ferric and ferrous chloride salts were obtained from Sigma (St. Louis, MO, USA). The initiator 2, 2´ azobisisobutyronitrile AIBN (Switzerland, Fluka) was recrystallized from ethanol at 50°C before use. Ammonium hydroxide NH_3_·H_2_O (30% w/v), Zn(NO_3_)_2_.4H_2_O, Na_2_CO_3_, potassium hydroxide 85%, carbon disulfide 99.5%, diethyl ether 99.5%, hydrochloric acid 32%, methyl cellulose, acetone 99.5%, THF, dimethylformamide 99.5% were obtained from Merck Chemical Co. DOX salts were obtained from Sobhan Pharmaceutical Co. (Tehran, Iran), and other biological reagents were purchased from Invitrogen Corp.

### Synthesis of magnetite nanoparticles

Magnetite nanoparticls (MNPs) were synthesized via improved chemical co-precipitation process.^32,33^ According to this technique, FeCl_3_.6H_2_O (7.57 g, 0.028 mol) and FeCl_2_.4H_2_O (3.17, 0.016 mol) were dissolved in 340 mL of double distilled water and stirred under nitrogen atmosphere at 75°Cfor 1 hour. Then, 40 mL of NH_4_OH (30% w/v) was quickly added to mixture under intense stirring. The solution was maintained at 80 ± 5°C for 1 hour. Then, reaction was cooled and obtained black nanoparticles was collected by centrifugation (13 000 rpm, 15 minutes) and washed four times with water and ethanol until its pH becomes absolutely neutral. Finally, black nanoparticles were dried under vacuum at 25°C temperature for 24 hours.

### Synthesis of ZnO nanoparticles

ZnO nanoparticles were obtained by a precipitation method.^34,35^ 0.1 mol aqueous solution of Zn (NO_3_)_2_.4H_2_O was added dropwise to 0.12 mol aqueous solution of Na_2_CO_3_ under vigorous stirring. The obtained white precipitates were centrifuged (13 000 rpm, 15 minutes) and washed with double distilled water several times. The solids were then washed with ethanol and dried at 100°C for 6 hours. Lastly; ZnO nanoparticles were achieved by calcination in the air at 250°C for 2.5 hours.

### Synthesis of acrylate grafted magnetic nanoparticles

**Figure 1:**
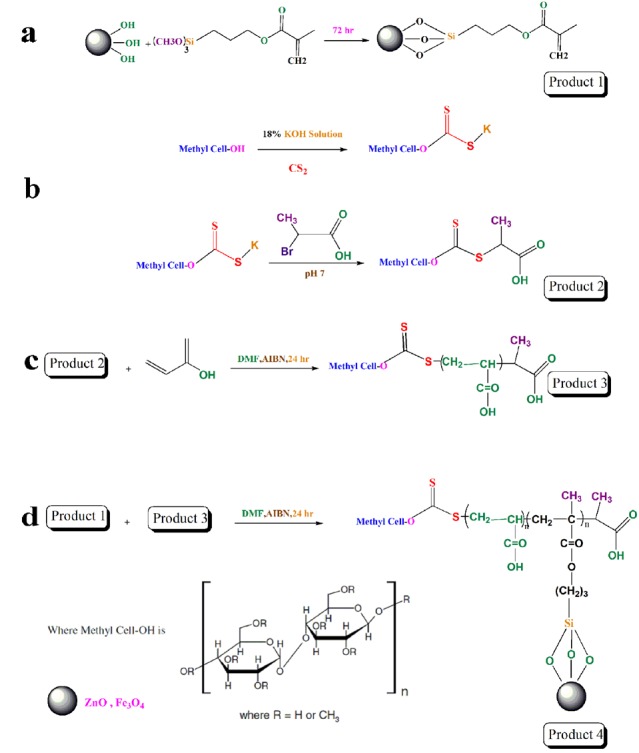
Synthesis of 3-(Trimethoxysilyl) propyl methacrylate (TSPMA) coated magnetic or ZnO nanoparticles (a), synthesis of propionic acid methyl cellulose xanthate RAFT agent (b), synthesis of Cellulos-g-PAA (c), synthesis of (Cellulos-g-PAA-g-PAcMNPs) or (Cellulos-g-PAA-g- PAcZnO) (d).

MNPs were activated in NaOH (1M) solution for 24 hours and washed with deionized water until the pH of the solution converted to neutral. Then the activated nanoparticles were vacuum dried at ambient temperature for 48 hours. Then Modified MNPs were dispersed in toluene with the aid of probe-type sonicator (UP 200H, Hielscher, Germany) for 5 minutes. 500 µL of TSPMA were added to 1 g of prepared MNPs dispersion in toluene in order to introduce the acrylate groups on the surface of MNPs. The dispersion was stirred for 24 hours and then AcMNPs were separated from unbounded TSPMA by an external magnet. The synthesis route is shown in Figure 1a.

### Synthesis of acrylate grafted ZnO nanoparticles

The procedure used to synthesize AcZnO materials was as same as the protocol that mentioned for the synthesis of AcMNPs. The only difference was that using of ZnO nanoparticles instead of MNPs. AcZnO materials were separated from unbounded TSPMA using the Amicon^®^ centrifugal filters (Ultra-15, molecular weight cutoff of 100 kDa, Millipore, Darmstadt, Germany) at 5000 rpm for 15 minutes (Figure 1a).

### Synthesis of propionic acid methyl cellulose xanthate RAFT agent

The protocol of propionic acid methyl cellulose xanthate RAFT agent synthesize was described previously.^16^ A 100 mL round-bottom flask was charged with 18% aqueous potassium hydroxide solution (30 mL), methylcellulose (MC, 2.5 g, 0.05 mmol). The mixture was stirred for 30 minutes at ambient temperature. At the end of this time, carbon disulfide (30 mL) was added during 10 minutes to the flask. And the resultant orange-colored solution was agitated for 7 hours at ambient temperature. At the end of this time the mixture was strongly agitated for 30 min with distilled water (50 mL). The extremely viscous orange solution of xanthate salt was precipitated in a mix of ether: cold methanol 1:1 (600 mL). The resultant yellow xanthate product was dissolved in ice-cold distilled water (75 mL). potassium hydroxide solution (1.12 g, 0.02 mol) with neutralized 2-bromopropionic acid (4 g, 2.35 mL) in distilled water (2 mL), was slowly added over 20 minutes at ambient temperature and stirred overnight.

By dropwise addition of 1M hydrochloric acid to the reaction mixture (pH to pH 2–3) precipitation happened immediately. Propionic acid hydroxypropyl cellulose xanthate RAFT agent was rinsed several times with ether and dried under vacuum at room temperature to afford yellow powder (Figure 1b).

### Synthesis of Cellulose-g-PAA

Acrylic acid (1.44 g, 20 mmol), AIBN (2.0 mg, 12 µmol), modified cellulosic materials (0.1 g) and dried dimethylformamide (7 mL) were mixed in a 25 mL round bottom flask. The reaction was done at 70 ± 3°C under an argon atmosphere with continuous stirring for about 24 hours. The obtained polymer was diluted with the addition of 9 mL DMF, and then purified by precipitation in cold diethyl ether. The obtained polymer was vacuum dried at 25°C for 24 hours (Figure 1c).

### Synthesis of Cellulose-g-PAA-g-PAcMNPs

In a 25 mL round bottom flask, AIBN (2.0 mg, 12 µmol), (AcMNPs) (1.5 g) and Cellulose-g-PAA (0.1g) were dissolved in DMF (4 mL) and the reaction was done at 75°C under an argon atmosphere for about 24 hours. Then 6 mL DMF was added to resulting polymer and the product was purified by precipitation in chilled diethyl ether (200 mL). The achieved nanocomposite was then vacuum dried at 25°C for 24 hours (Figure 1d).

### Synthesis of Cellulose-g-PAA-g-PAcZnO

The procedure used to synthesize Cellulose-g-PAA-g-PAcZnO was as similar as the method mentioned for the synthesis of Cellulose-g-PAA-g-PAcMNPs. The only difference was that using of ZnO nanoparticles instead of MNPs (Figure 1d).

### Preparation of DOX-loaded nanocarriers

Appropriate amount of Cellulose-g-PAA-g-PAcZnO and Cellulose-g-PAA-g-PAcMNPs nanocarriers was ultrasonically spread in predetermined amount of DOX solution and keep in dark condition under stirring for overnight. DOX-loaded nanocarriers were separated from unloaded DOX using the Amicon^®^ centrifugal filters (Ultra-15, molecular weight cutoff of 100 kDa, Millipore, Darmstadt, Germany) at 5000 rpm for 15 minutes. Finally, DOX-loaded nanocarriers were freeze-dried. DOX loading was measured using UV-Vis spectrophotometer at a wavelength of 470 and 285. Drug encapsulation efficiency (EE) and drug loading capacity of DOX was calculated as follows:

$$\mathrm{LC(\%)=}\frac{\mathrm{milligram of drug in nanomicelle}}{\mathrm{milligram of nanomicelle}}\times 100$$

$$\mathrm{EF(\%)=}\frac{\mathrm{milligram of drug in nanomicelle}}{\mathrm{milligram of initial added drug}}\times 100$$

### In vitro DOX release

DOX release was investigated according to the following method. In 2 mL PBS solutions with two pH values (5.4, and 7.4), 5 mg of DOX-loaded nanocarriers was dispersed using bath sonicator for 1 minute. The samples were then placed in an incubator at 37°C for certain time intervals. To measure the amount of drug released, samples were collected by centrifugation and the supernatant was taken out at predetermined time intervals, then the same volume of fresh PBS was added. The absorbance of released DOX was measured by a UV-Vis spectrophotometer and converted to concentration by interpolating the absorbance of DOX to their calibration curves separately at pH values of 5.4 and 7.4. The drug release experiments were done in triplicate, and the average of the data was reported.

$$\text{Drug release(\%)}=\frac{\text{ammount of drug release}}{\text{amount of drug on nanocarrier}}\times 100$$

### In vitro cytotoxicity studies

The relative cytotoxicity of nanocarriers, free DOX, and DOX-loaded nanocarriers were assessed against the MCF7 after 48 hours exposure time by MTT assay method based on the following method.^12,36^ The MCF7 cells were cultured in Roswell Park Memorial Institute 1640 growth medium (RPMI 1640 medium; Gibco BRL Life Technologies) containing 10% fetal bovine serum, 100 U/mL penicillin and 100 µg/mL streptomycin and incubated at 37°C in an atmosphere of 5% CO2 and 95% air with more than 95% humidity in the incubator to allow the cells to grow and attach to the bottom of each well. Then the medium was changed with fresh culture growth medium having DOX and DOX-loaded nanocarriers with varying concentrations of the drugs (25, 50, 100 and 200 µg/mL). Furthermore, for biocompatibility evaluation of the novel nanocarriers, different concentrations of Cellulose-g-PAA-g-PAcMNPs and Cellulose-g-PAA-g- PAcZnO were used. Cancer cells without any treatment were used as a negative control. The MTT assay was done as follows: after 48 hours incubation, the cell media were omitted and the cells were gently washed with sterilized PBS solution. MTT solution (5 mg/mL) was added to each well and incubated for an additional four hours. After removing MTT solution from each well, DMSO and Sorenson buffer was added to each well for dissolving the produced blue formazan crystals and absorbance of solubilized formazan was determined by an ELISA plate reader (Awareness Technology, Palm City, FL, USA) at 570 nm with a reference wavelength of 630 nm, and the growth inhibition was evaluated. All tests were done in triplicate. The relative cell viability (%) was calculated using the following equation:

$$\text{Cell Viability(\%)=}\frac{A_{\mathrm{test}}}{A_{\mathrm{control}}}\times \text{100}$$

Where A_test_ and A_control_ are the mean absorbance values of the tested groups and control groups (without any treatment), respectively

### DAPI staining

DAPI staining was done for visualization of the condensed and fragmented nuclei of the apoptotic cells induced by nanoparticles (nanocarriers, free drugs, and DOX-loaded nanocarriers).^37^ MCF7 cells were seeded into slide chambers and incubated at 37°C for 24 hours. After the cells were grown in culture media, the medium was exchanged by fresh media having nanocarriers (1000 µg/mL) as well as free drugs and drug-loaded nanocarriers in which the concentrations of the DOX was 100 µg/mL. After 48 hours, the cells were washed with fresh PBS (pH 7.4), fixed with paraformaldehyde (4 wt%, 10 minutes), permeabilized with Triton X-100 (0.1% w/v, 15 min) and stained with DAPI (300 ng mL^−1^, 5 minutes). DNA condensation and fragmentation in apoptotic cells were evaluated under a fluorescence microscope (Olympus microscope Bh2-RFCA, Japan). All tests were performed in duplicate.

### Instrumentation

Proton nuclear magnetic resonance (^1^HNMR) spectra were tested at 25°C via ^1^HNMR (400 mHz) Bruker spectrometer (Bruker, Ettlingen, Germany). Fourier transform infrared (FT-IR) spectra of the samples were recorded by Shimadzu 8101M FT-IR (Shimadzu, Kyoto, Japan) at the wavenumber ranges of 400 to 4000 cm^–1^. Laser-scattering technique (Zetasizer Nano ZS90; Malvern Instruments, Malvern, UK) were used to measure the average diameter at 25°C. A vibrating sample magnetometer (VSM; AGFM, Kashan, Iran) was used to probe the magnetic properties of nanoparticles at ambient temperature. The surface morphology and size of nanocarriers were observed by a field emission scanning electron microscope-energy dispersive X-ray (FESEM-EDX; S4160 Hitachi, Japan).

### Statistical analysis

We performed a statistical analysis on qRT-PCR results using the Student *t* test. The p-values were considered significant when they dropped down to ≤0.05.

## Results and Discussion

**Figure 2:**
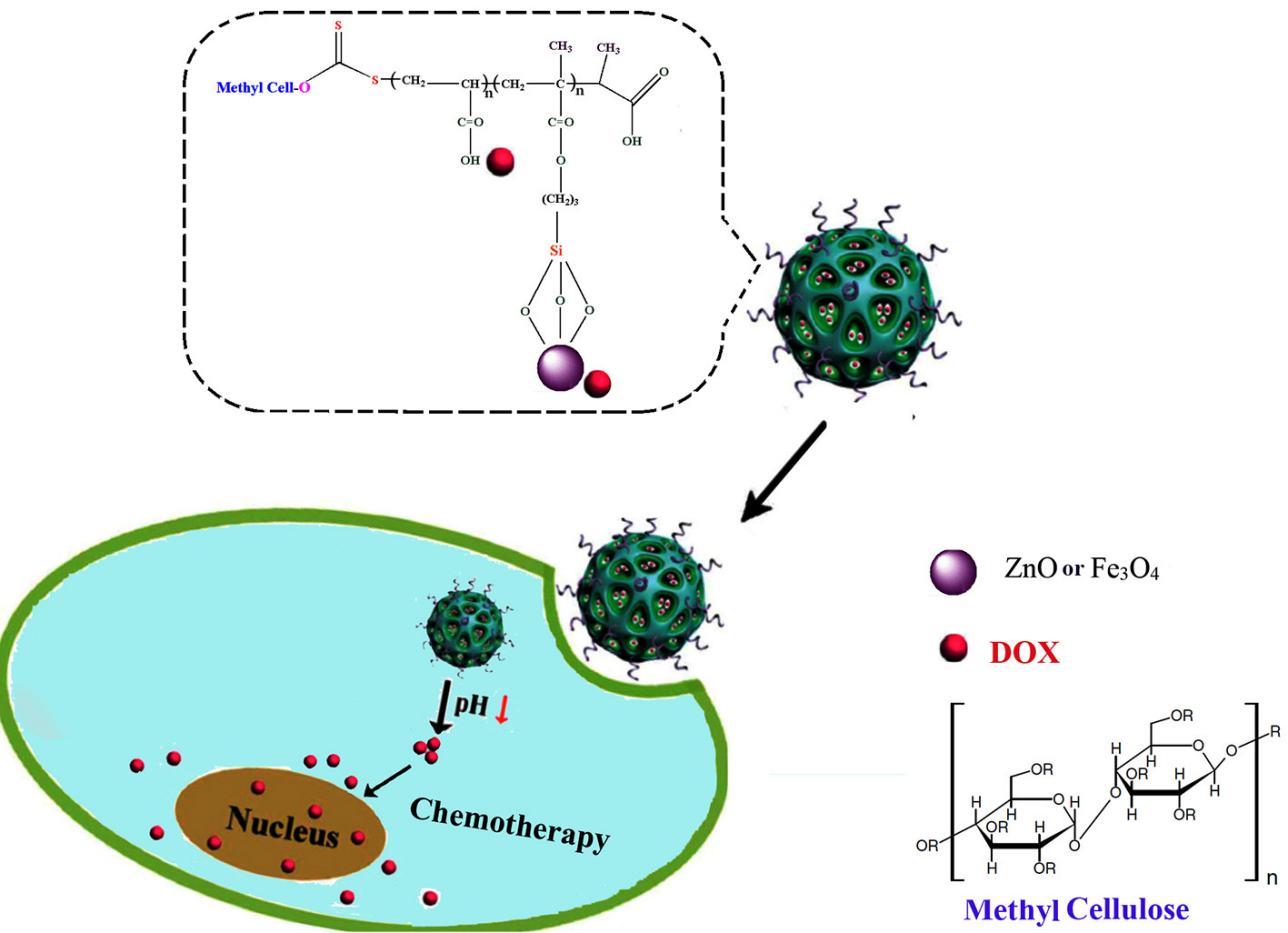
Cell uptake mechanism of the nanocarrier-DOX complex by MCF7 cells

Polysaccharide-based nanoparticulate drug delivery systems (PDSs) can be seen as a powerful tool to overcome the limitations of traditional chemotherapy. PDSs offer many advantages to traditional chemotherapy by manipulating the properties of the drug. For example, conjugating a small molecule drug to a polymer can protect it from systemic degradation, and increase stability in the bloodstream. According to this facts, we have developed smart nanocarriers based on cellulose with highly carboxylic acid functional groups, good biocompatibility, pH-controlled DOX release behavior, and efficient anticancer efficacy on breast cancer cells (Figure 2).

### Characterization of Cellulose-g-PAA

**Figure 3:**
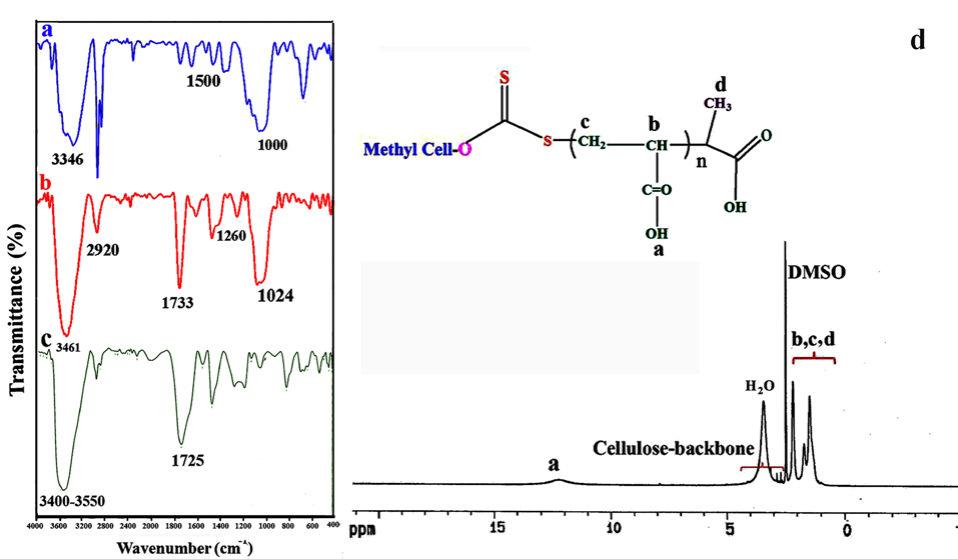
FTIR spectra of methyl cellulose (a), propionic acid methyl cellulose xanthate RAFT agent (b), Cellulos-g-PAA (c), ^1^H NMR spectra of Cellulos-g-PAA (d).

Cellulose-g-PAA was synthesized through the polymerization of acrylic acid directly onto the propionic acid methyl cellulose xanthate RAFT agent as described in experimental section. The synthesized Cellulose-g-PAA was characterized using FTIR and ^1^H NMR spectroscopies. Figure 3 showed FTIR and HNMR spectrum of the cellulosic materials before and after modification. A broad peak in 2480-3410 cm^-1^ distances appears due to celluloses active OH groups stretching vibration. Stretching vibration in 1000 cm^-1^ is related to C-O group and existing peak in 1500 cm^-1^ distance is related to cellulose rings C-C absorption band (Figure 3a).

Figure 3b shows, RAFT reagent connected to the cellulosic backbone. Its RAFT spectrum illustrates a broad peak in 3200-3800 cm^-1^ related to vibration of cellulose OH groups, a peak in 2920 cm^-1^ distance related to stretching vibration of C-C simple bond, stretching vibration of carbonic groups (C=O) in 1733 cm^-1^, and a clear peak in 1024 cm^-1^ related to C=S double bond which proves the truth of xanthate reagent formation.

Figure 3c shows, a very strong band for a carbonyl C=O group at 1725 cm-1 correspond to graft copolymers and modified cellulosic compounds (Figure 3b). This confirmed the successful polymerization of acrylic acid on the cellulosic materials.

The ^1^HNMR spectra of the synthesized Cellulose-g-PAA are revealed in Figure 2d. The chemical shifts at 3.00 to 4.50 ppm corresponded to the cellulose backbone. The incorporation of polyacrylic acid into the cellulose backbone is confirmed by the presence of chemical shift at 0.5-2.10 ppm and 12 ppm related to the –CH_2_, –CH_3_,–CH groups and –OH protons of hydroxyl respectively.

### Characterization of nanocarriers

The synthesized Cellulose-g-PAA-g-PAcZnO and Cellulose-g-PAA-g-PAcMNPs nanocomposite were characterized by means of FTIR, TGA, VSM, dynamic light scattering (DLS), scanning electron microscope (SEM)/EDX spectroscopes.

The FTIR spectra of the mentioned samples are shown in Figure 3a. FT-IR spectra of ZnO sample show a dominant ZnO absorption band between 435 and 528 cm^−1^. The broad peak at 3600 cm^−1^ is because of the stretching vibrations of the –OH group on the surface of ZnO nanoparticles.

**Figure 4:**
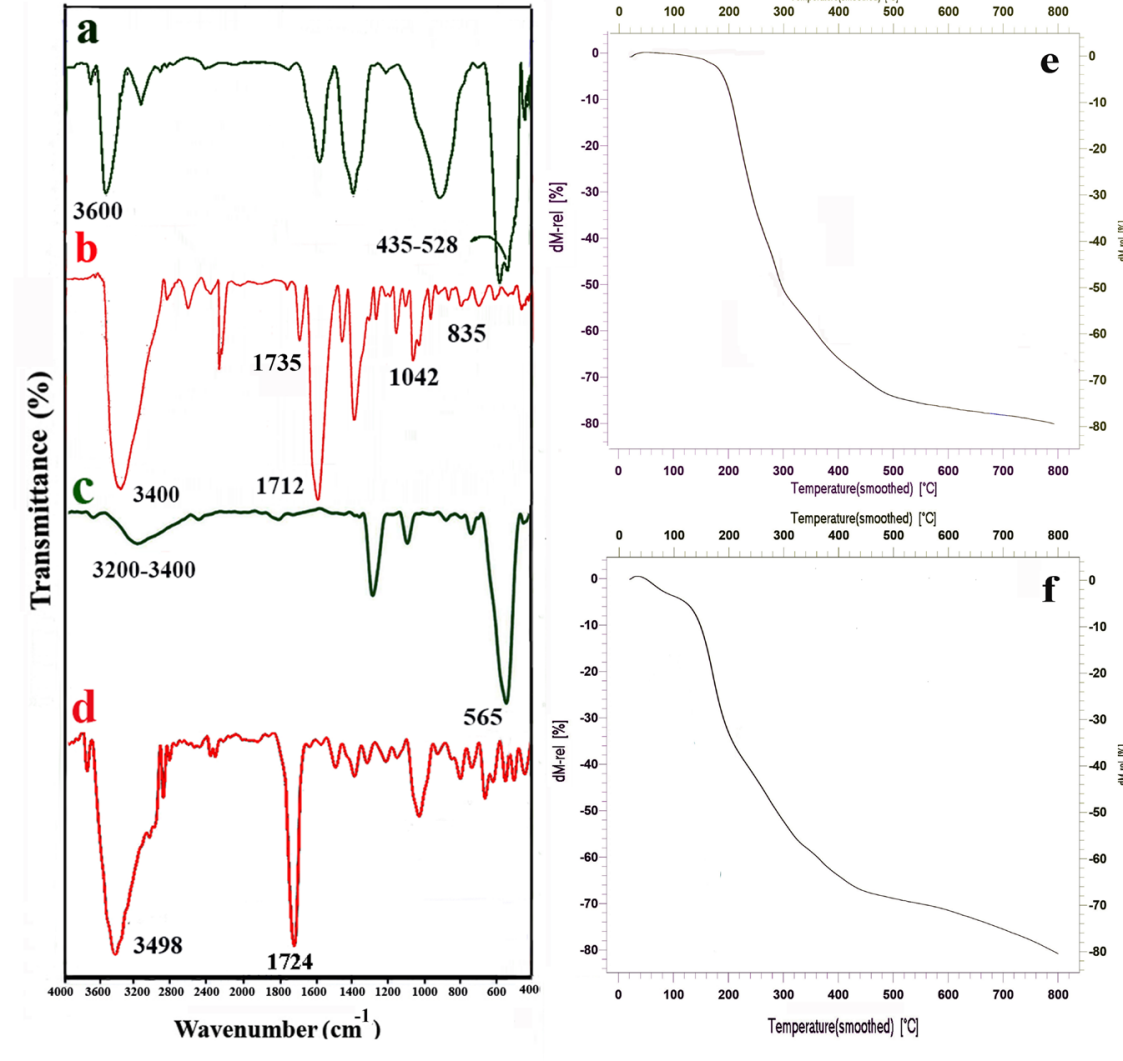
FTIR spectra of ZnO nanoparticle (a), Cellulos-g-PAA-g-PAcZnO (b), Fe_3_O_4_ (MNP) (c) and Cellulos-g-PAA-g-PAcMNPs (d), TGA curves of Cellulos-g-PAA-g-PAcMNPs (e) and Cellulos-g-PAA-g-PAcZnO (f).

Cellulose-g-PAA-g-PAcZnO nanocomposite (Figure 4b) exhibited the characteristic bands related to all composite segments. Introduction of TSPMA to the surface of nanoparticles (ZnO) is confirmed by the bands at 1042 cm^−1^ assigned to the Si–O stretching vibrations and 835 cm^−1^ assigned to the Zn-O-Si. The broadband at 3400 cm^−1^ is referred to the OH stretching vibration of polyacrylic acid and the peak at 1712 and 1735 cm^−1^ shows carbonyl groups of PAA and TSPMA, respectively.

In the FT-IR spectrum of the Fe_3_O_4_ nanoparticles (Figure 4c), the peaks at 565 and 3417 cm^-1^ were related to the stretching vibrations of Fe-Oand OH, respectively.

The FTIR spectrum of the Cellulose-g-PAA-g-PAcMNPs nanocomposite is shown in Figure 4d. The appearance of the stretching vibrations of C=O at 1724 cm^-1^ region related to PAA and TSPMA moiety of the nanocomposite.

The thermal decomposition of the Cellulose-g-PAA-g-PAcMNPs (Fe_3_O_4_-nanocarrier) and the Cellulose-g-PAA-g-PAcZnO (ZnO-nanocarrier) were surveyed using the TGA method (Figure 4e-4f)). Both compounds had three-stage decomposition profiles. The first step of weight loss which appeared below 150°C concerned the removal of solvent or moisture and degradation of small molecules (3% for Fe_3_O_4_-nanocarrier and 9% for ZnO-nanocarrier). The second stage of destruction which appeared at 150–450°C related the decomposition of polyacrylic acid and the separation of the organic groups attached to the main polymer chains (66.6% for Fe_3_O_4_-nanocarrier and 59% for ZnO-nanocarrier. The third stage occurred at temperatures between 450 and 800°C and is attributed to the decomposition of cellulose backbone (12% for Fe_3_O_4_-nanocarrier and 15% ZnO-nanocarrier). The total weight loss for the Fe_3_O_4_-nanocarrier at 800°C was 81.66% and for ZnO-nanocarrier was 83%. The residual part in the TGA thermogram of nanocomposite that did not burn with temperature up to 800°C, was showed the remaining Fe_3_O_4_ and ZnO particles.

**Figure 5:**
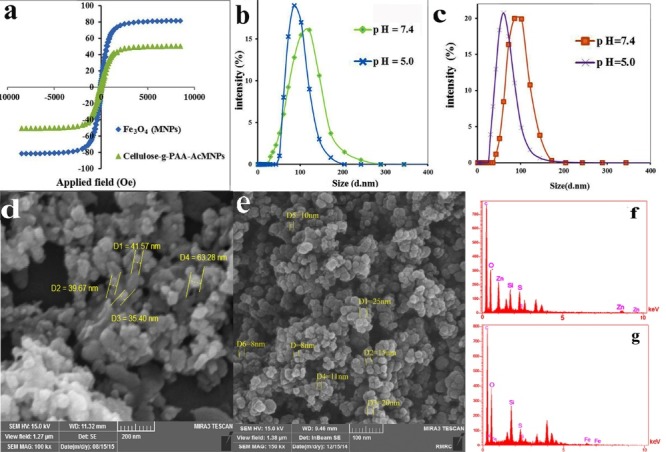
Magnetic properties of Fe_3_O_4_, and Cellulos-g-PAA-g-PAcMNPs with magnetization values of about 81 and 50 respectively (a) Hydrodynamic diameters of Cellulos-g-PAA-g-PAcZnO (b), Cellulos-g-PAA-g-PAcMNPs (c), Surface morphology of Cellulos-g-PAA-g-PAcZnO (d), Cellulos-g-PAA-g-PAcMNPs (e) Semi-qualitative determination of elements on the surface of Cellulos-g-PAA-g-PAcZnO (f) , Cellulos-g-PAA-g-PAcMNPs (g) by SEM-EDX elemental analysis.

The magnetic properties of Fe_3_O_4_ nanoparticles, and Cellulose-g-PAA-g-PAcMNPs nanocarrier were measured by a VSM at 298 K in a field of 10 kOe (Figure 5 a). As shown by the results, Fe_3_O_4_ NPs have superparamagnetic behavior and the magnetization value at 10 kOe was about 50 emu g^−1^. Its saturation magnetization amounts were fewer than that of bulk Fe_3_O_4_ (as a reference value, 81emu g^−1^), because the surface of Fe_3_O_4_ NPs was coated with polymeric materials. The magnetization results showed that the magnetic nanocarrier has a superparamagnetic nature.

The particle size distribution of Cellulose-g-PAA-g-PAcMNPs and Cellulose-g-PAA-g-PAcZnO nanocarriers at two different pH values were determined by DLS studies (Figure 5 b,c ). Generally, the nanoparticles with potential for aggregation showed bigger average hydrodynamic particle sizes, compared to that obtained by SEM. Indeed, as the nanocarrier is dispersed in PBS, the different surface charges on the nanocarrier cause slight aggregation. In addition, DLS analysis showed the hydrodynamic diameter of the nanoparticles in aqueous solution; however, SEM indicated the diameter of dried nanocarriers. However, the both nanocarriers with highly functional groups showed negligible aggregation in PBS at two pH values (5.4 and 7.4), with average hydrodynamic sizes of approximately 85.8 and 121.2 nm for Cellulose-g-PAA-g-PAcZnO and average hydrodynamic sizes around 60.8 and 102.2 nm for Cellulose-g-PAA-g-PAcMNPs nanocarrier, respectively. As the results, the lower hydrodynamic size of the nanocarrier at pH 5.4, compared to pH 7.4, is attributed to the hydrogen bonding within the protonated PAA, which cause decreasing hydrodynamic diameters in the nanocarrier at low pH values. All observations indicate that the synthesized nanocarriers were pH-responsive and could be used as smart. All observation indicate that the Cellulose-g-PAA-g-PAcMNPs and Cellulose-g-PAA-g-PAcZnO could use as pH-responsive nanocarriers in DDS.

SEM showed a definite appearance of the morphology of the nanocomposite. Cellulose-g-PAA-g-PAcZnO (Figure 5d) and Cellulose-g-PAA-g-PAcMNPs nanocomposite (Figure 5e) shows spherical shape, smooth surface and uniform size distribution, and the mean particles size were 38 and 15 nm respectively.

The presence of O, Fe, C, Zn, S and Si elements were determined semi-qualitatively on the surface of nanocarriers by selected area energy dispersion spectrum (Figure 5f,d). From the EDX results calculation and TGA curves evaluation, organic parts percentage of Cellulose-g-PAA-g-PAcMNPs were obtained around 97.39% and 81.66%, respectively. However, the evaluation of the fraction of organic parts for Cellulose-g-PAA-g-PAcZnO which extracted from EDX results and TGA curves became approximately 95.39% and 83%. The results obtained from both approaches were approximately similar and approved each other. These results indicate that the nanocarriers synthesized successfully.

### In vitro DOX loading, release study and Cytotoxicity assay

DOX was well soluble in PBS; therefore its attachment to the nanocarriers proceeded easily. Unbound DOX could be removed from the surface of the Cellulose-g-PAA-g-PAcMNPs by the use of an external permanent magnet as well as, for Cellulose-g-PAA-g-PAcZnO by the use of centrifugation and washing several times with PBS. The obtained supernatants were removed for the evaluation of the encapsulation and loading efficiency of DOX. The encapsulation efficiency of DOX on Cellulose-g-PAA-g-PAcZnO and Cellulose-g-PAA-g-PAcMNPs was 99.07% and 98.92%, respectively. Also DOX loading capacity of Cellulose-g-PAA-g-PAcZnO and Cellulose-g-PAA-g-PAcMNPs was 90% and 89.09%, respectively. The in vitro DOX release behavior from the nanocarriers were determined in PBS at pH 7.4, 37°C and pH 5.4, 40°C at which represent the environment of blood and the tumor microenvironment, respectively.

**Figure 6:**
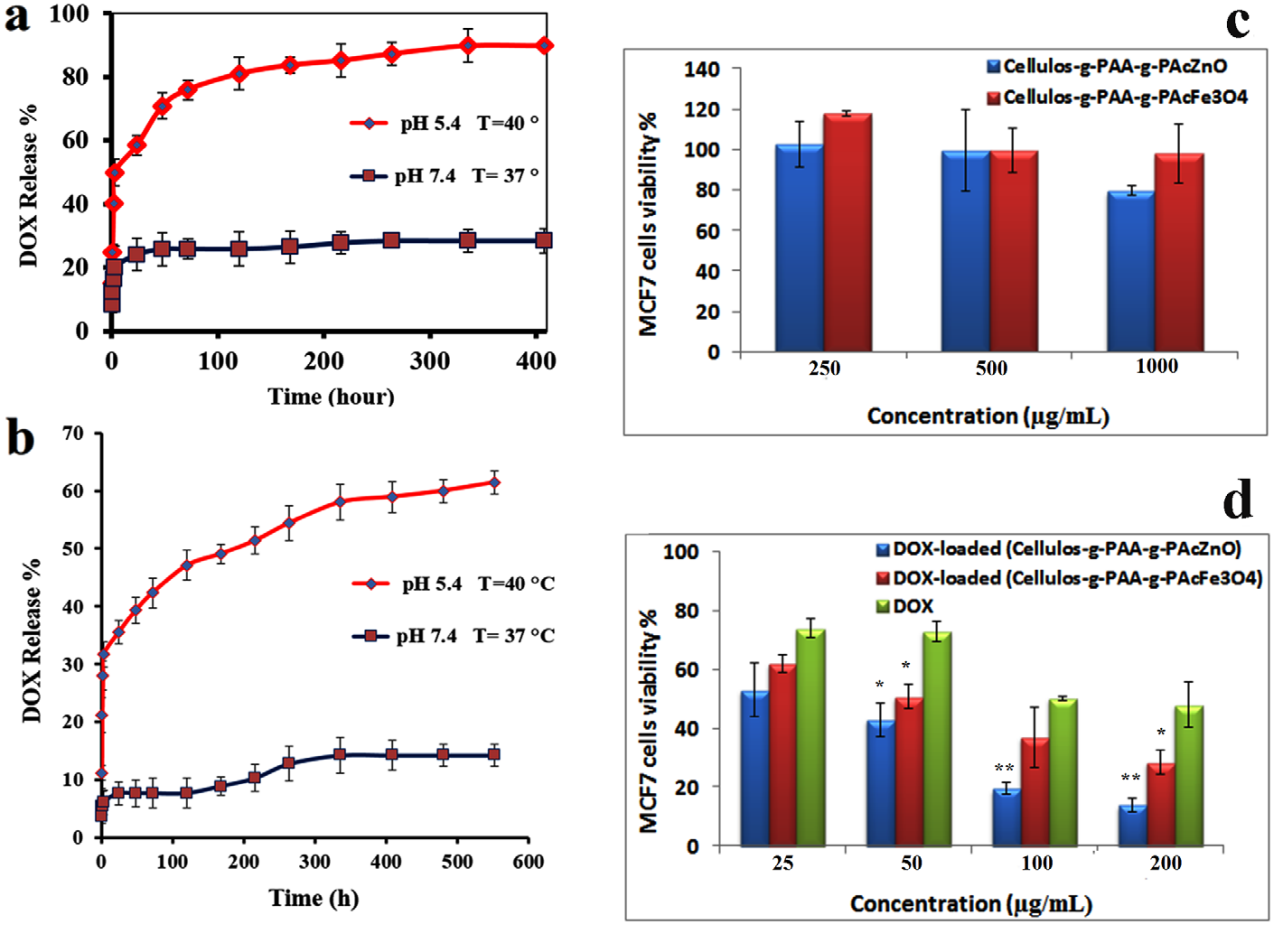
*In vitro* release profiles of DOX loaded Cellulos-g-PAA-g-PAcMNPs (a) DOX loaded Cellulos-g-PAA-g-PAcZnO (b) at pHs 5.4, 40°C and pH 7.4, 37°C, Cell growth inhibition rates by different concentrations of free DOX, DOX@ nanocarrier, and nanocarrier after 48 h exposure with the MCF7 cell line (c,d).

The release profile for both nanocarriers (Figure 6a,b) was considered at three phases: burst release at the first 2 hours (period I), followed by fast release from 2 to 300 hours (period II), and finally constant release from 300 to 552 hours (period III). High concentration gradient among the release medium and the nanocarriers surface which performed as the motivating power for drug transmission at the first 2 hours. Moreover, the physically absorbed DOX on nanocarriers was released in this step. Between 2 and 300 hours (period II), the pH-dependent release from nanocarriers became dominant (89% pH = 5.4, 28% pH=7.4 from Cellulose-g-PAA-g-PAcMNPs and 58% pH= 5.4, 14% pH =7.4 from Cellulose-g-PAA-g-PAcZnO were released in this stage). It was expected that the major part of DOX-loaded nanocarrier started to release in stage II. The continuous release profile was gained at 300–552 hours. The kinetics of DOX release from controlled release Cellulose-g-PAA-g-PAcZnO nanoformulation at pH 5.4 (maximum RSQ:0.942, minimum MPE:8.503) and 7.4 (maximum RSQ:0.928, minimum MPE:10.444) obeys Weibull and Higuchi model, respectively. Also the kinetics of DOX release from Cellulose-g-PAA-g-PAcMNPs at pH 5.4 (maximum RSQ:0.979, minimum MPE:6.19) and 7.4 (maximum RSQ:0.874, minimum MPE:9.233) obtained log-probability.

The formation of the complexes between the DOX and ZnO nanoparticles at the chelating sites of the quinone and the phenolic oxygen molecules on both sides of the DOX aromatic moiety, on the one hand, and ionic interactions between DOX and polyacrylic acid affected the alterations in the release profiles.

At mild acidic pH, a noticeable growth in the drug release rate was detected. Because, the protonation of the carboxyl group of PAA under acidic conditions (pka=6-6.5) lead to faster detachment of the DOX-loaded nanocarriers, leading to more DOX release at lower pHs. As well as, at neutral pH (7.4), the fully deprotonated PAA resulted in a strong interaction with DOX, which delayed the release of a loaded drug. Also, the sustainer DOX release from Cellulose-g-PAA-g-PAcZnO nanoparticles compared to Cellulose-g-PAA-g-PAcMNPs was due to the formation of a strong complex between ZnO and chelating sites of the DOX that hinder the DOX release.

Drug release studies confirmed that novel developed DOX-loaded nanocarriers were a suitable nominee for cancer therapy because it keeps a low drug release rate in the simulated bloodstream under physiological situations (pH 7.4 and 37°C) though having a great drug release rate under cancerous situations (pH 5.4 and 40°C).

In vitro cellular cytotoxicity studies of the DOX, DOX-loaded nanocarriers, and free nanocarriers were evaluated by MTT assay after 48 hours exposure with MCF7 cell lines (Figure 6c,d). MCF7 cell line which received DOX free nanocarriers, at a concentration of ten times greater than the DOX concentration, showed no considerable cytotoxicity. Furthermore, to compare the cytotoxicity effects of both free drug and drug-loaded nanocarriers, the same concentration of drugs was used for all treatment times. The IC_50_ of the DOX loaded (Cellulose-g-PAA-g-PAcZnO) and DOX loaded (Cellulose-g-PAA-g-PAcMNPs) after 48 h treatment with MCF7 cell lines were about 24.03 and 49.27 μg mL^−1^, respectively, whereas the IC_50_ of the free DOX was 99.76 μg mL^−1^, respectively. Cell toxicity study revealed that in DOX nanoformulation, the DOX constantly released in its active form without losing cytotoxicity properties. Therefore the limitations of this chemotherapy drug, like short half-life in the human body could be prevail by incorporating it into the nanocarriers. As a result, active treatment of MCF7 cells could be attained with lengthier exposure time and minor DOX concentration.

**Figure 7:**
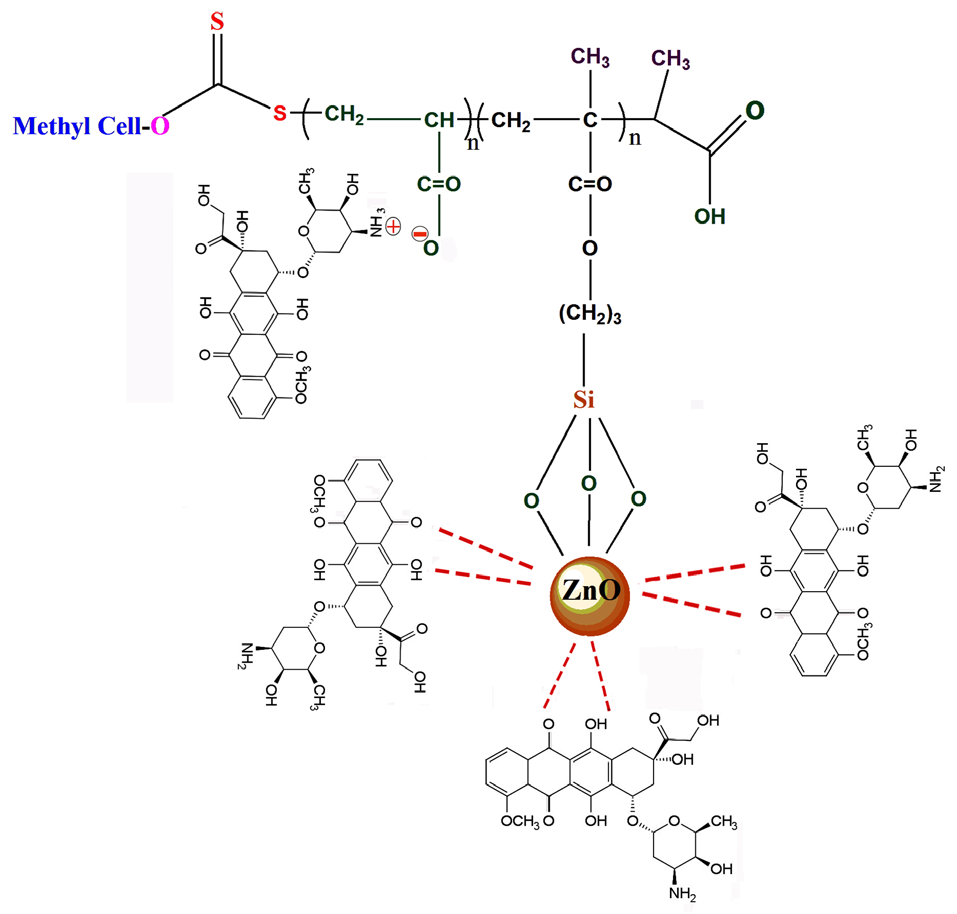
Schematic representation of DOX loaded (Cellulose-g-PAA-g-PAcZnO) NPs.

Furthermore, DOX-loaded Cellulose-g-PAA-g-PAcZnO shows higher chemotherapy efficiency compare to the DOX-loaded Cellulose-g-PAA-g-PAcMNPs due to high interaction of ZnO with DOX. The formation of the complexes between the DOX and ZnO nanoparticles at the chelating places of the quinone and the phenolic oxygen molecules, enhanced chemotherapy effectiveness by additional intracellular concentration of DOX (Figure 7).^38,39^

### Induction of apoptosis by DAPI staining

**Figure 8:**
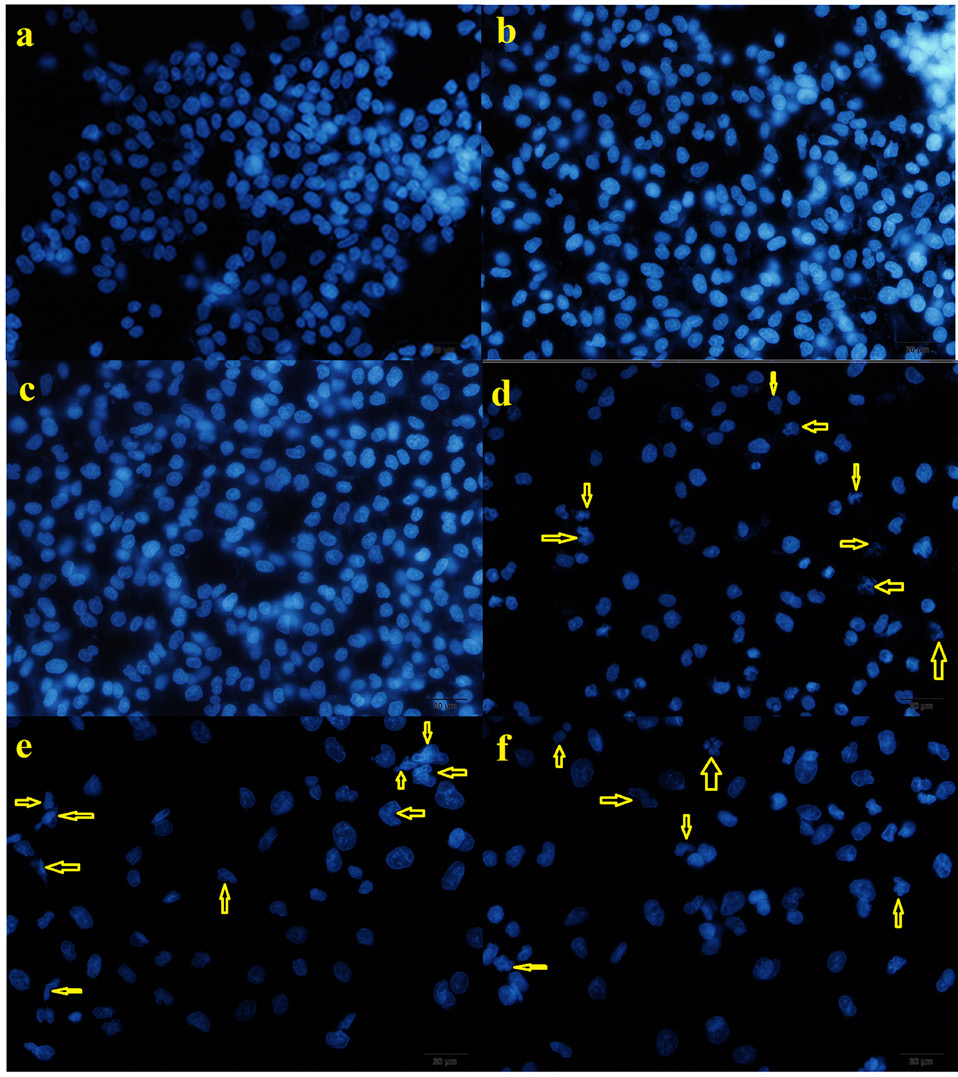
Microscopic images of DAPI stained MCF7 cells following 48 h of exposure to untreated cells as a control (a), Cellulos-g-PAA-g-PAcZnO (b), Cellulos-g-PAA-g-PAcMNPs (c), free DOX (d), DOX loaded (Cellulos-g-PAA-g-PAcZnO) (e) and DOX loaded (Cellulos-g-PAA-g-PAcMNPs) (f).

To evaluate the normal, apoptotic, and necrotic MCF7 cells, the chromatin morphologies were studied by DAPI staining method. The MCF7 cells without treatment had no changes in nuclei and no necrosis of cancer cells was detected (Figure 8). The cells treated with drug-free nanocomposites did not show alterations in their chromatin appearance, whereas both the free DOX and DOX-loaded nanocarriers had high chromatin fragmentation. However, it was clear that the cells received the DOX-loaded nanocomposites showed more changes in the cell nuclei compared to free DOX with equal drug concentration and time.

## Conclusion

In this report, we designed polysaccharide-based ZnO and Fe_3_O_4_ nanoparticulate DOX delivery systems to increase anticancer efficacy of DOX in MCF-7 breast cancer cells. In this context, nanocarriers were synthesized via polymerization of acrylic acid with ZnO and Fe_3_O_4_ modified 3-(trimethoxysilyl) propyl methacrylate onto the cellulosic backbone via RAFT method. Furthermore, DOX was loaded effectively to the ZnO and Fe_3_O_4_ nanocarriers via complexing and electrostatic force with great loading efficiency (99.07% and 98.92%) respectively. Drug release studies showed the potential of DOX-loaded nanocarriers for cancer therapy due to their negligible DOX release rate under physiological situations (pH 7.4) and accelerated DOX release rate under cancerous conditions (pH 5.4). MTT assay and DAPI staining were shown that DOX-ZnO nanocarrier shows higher chemotherapy efficiency compare to the DOX-Fe_3_O_4_ nanocarrier due to the high chelating of ZnO with DOX. Based on these results, DOX-loaded nanocarriers with synergistic effects revealed considerably enhanced curative effect and holds hopeful prospects in the field of nanomedicine.

## Ethical Issues

Not applicable.

## Conflict of Interest

Authors confirm that they have no conflict of interest.

## Acknowledgments

This study was financially supported by Drug Applied Research Center of Tabriz University of Medical Sciences.
